# Diagnosis of Whipple’s disease with pseudorheumatoid nodules in a patient treated with biologics for rheumatoid polyarthritis

**DOI:** 10.1093/rap/rkad002

**Published:** 2023-01-10

**Authors:** Marie Doussiere, Jean-Marc Sobhy Danial, Clémence Barthomeuf, Jean-René Tesson, Quentin Beauvillain, Vincent Goeb

**Affiliations:** Service de Rhumatologie, Université de Picardie Jules Verne, CHU Amiens-Picardie, Amiens, France; Service de Rhumatologie, Université de Picardie Jules Verne, CHU Amiens-Picardie, Amiens, France; Service d’Anatomie et Cytologie Pathologiques, Université de Picardie Jules Verne, CHU Amiens-Picardie, Amiens, France; Service d’Anatomie et Cytologie Pathologiques, Université de Picardie Jules Verne, CHU Amiens-Picardie, Amiens, France; Service de Dermatologie, Université de Picardie Jules Verne, CHU Amiens-Picardie, Amiens, France; Service de Rhumatologie, Université de Picardie Jules Verne, CHU Amiens-Picardie, Amiens, France

Key message

- Whipple’s disease can take on deceptive forms and, in particular, hide in the form of polyarthritis with rheumatoid factors.

Dear Editor, A 65-year-old woman treated for RA with sarilumab and prednisone (10 mg/day) was hospitalized in September 2021 for bilateral ankle arthritis, fever and cachexia. RA was diagnosed in 2003, with the association of bilateral ankle arthritis, with CRP 10 mg/l and RF 330 IU/ml. ACPAs were negative. She had a medical history of psoriasis and severe glucocorticoid-induced osteoporosis, with three vertebral fractures treated with denosumab. She had not travelled abroad for >20 years. She had experienced episodes of bilateral ankle arthritis since 2003, with oedemas and hypodermitis on the lower half of the calves. She had subcutaneous, mobile and centimetric nodules of the left olecranon and the anterior right knee suggestive of rheumatoid nodules, associated with urticarial lesions of the thighs and forearms. These nodules had progressively increased in size and number since the onset of symptoms after treatment. Since 2016, she had daily episodes of hyperthermia between 38 and 39.5°C, with increased CRP, up to 200 mg/l in 2021. She had abdominal bloating, headache disorders, breathlessness and weight loss (7 kg). In July 2020, electromyogram performed for pain in the lower limbs revealed sensory and motor axonal neuropathy. A second electromyogram performed during her hospitalization in September 2021 was normal. Successive treatments with corticosteroids, synthetic DMARDs (leflunomide, methotrexate) and biologic DMARDs [bDMARDs; TNF inhibitors (infliximab, adalimumab, etanercept), abatacept and tocilizumab] were initiated without efficacy. From 2016 to 2018, while she was treated with rituximab, CRP increased to 100 mg/l. It was discontinued in May 2018 and replaced with baricitinib (anti-Janus kinase 2) with persistence of CRP associated with fever at 39.5°C. In December 2018, Still’s disease was suggested and treatment with anakinra 100 mg/day was tried, quickly stopped for allergic reaction and replaced by sarilumab, with persistence of increased CRP up to 200 mg/day and fever. In November 2020, it was replaced by upadacitinib, which was ineffective, with resumption of sarilumab in February 2021. There was good efficacy for fever and inflammatory syndrome (CRP 40 mg/l) after the resumption of sarilumab, without efficacy for other symptoms.

The patient was then referred to our department. RF was 81 UI/ml and ACPAs were negative during the hospitalization. Faecal calprotectin was 61.57 µg/g, ascorbic acid was <3 µmol/l, CA-125 was 446.2 U/ml and CA 15-3 was 35.8 U/ml. Haemocultures were negative. Whipple’s disease was diagnosed with detection of *Tropheryma whipplei* by positive PCR performed on blood, faeces, saliva, cutaneous biopsies and cerebrospinal fluid. There was no intra-articular effusion. Biopsies of deep skin urticarial lesions and duodenum, a lumbar puncture and the resection of two subcutaneous nodules were also performed for pathological examination. The deep skin biopsy showed a normal epidermidis, with septal dermohypodermatitis. In the dermis and hypodermis, there were many non-foamy macrophages, expressing CD68 in immunohistochemistry, whose cytoplasm contained multiple periodic acid–Schiff (PAS)-positive intracytoplasmic granulations (Fig. 1). These were positive to immunohistochemical examination with anti-*T. whipplei* antibody. Duodenum biopsies showed the same macrophages with PAS-positive intracytoplasmic granulations in the mucosae and the submucosae. These were also positive on immunohistochemical examination with anti-*T. whipplei* antibody. Gastric biopsies showed reactive gastritis, without macrophage infiltrate. Subcutaneous nodules revealed, for the first nodule, a typical angioleiomyoma surrounded by macrophages, with the same PAS-positive intracytoplasmic granulations, highly suggestive of *T. whipplei*. The second nodule was a reactive fibrotic and necrobiotic nodule containing many similar macrophages. Cytological analysis of the cerebrospinal fluid showed some lymphocytes and neutrophils, with some macrophages whose cytoplasm contained small bacilli, suggestive of *T. whipplei*. PAS staining and immunohistochemical examination could not be performed due to a lack of material available. X-rays of the ankles showed tarsitis. Endoscopy was normal. Chest CT found interstitial syndrome with mild pericardial effusion. Cardiac echography found multiple microcalcifications without endocarditis. Cardiac MRI showed stigmata of myocarditis. PET showed hypermetabolic infracentimetric mesenteric adenopathies.

**Figure 1.**
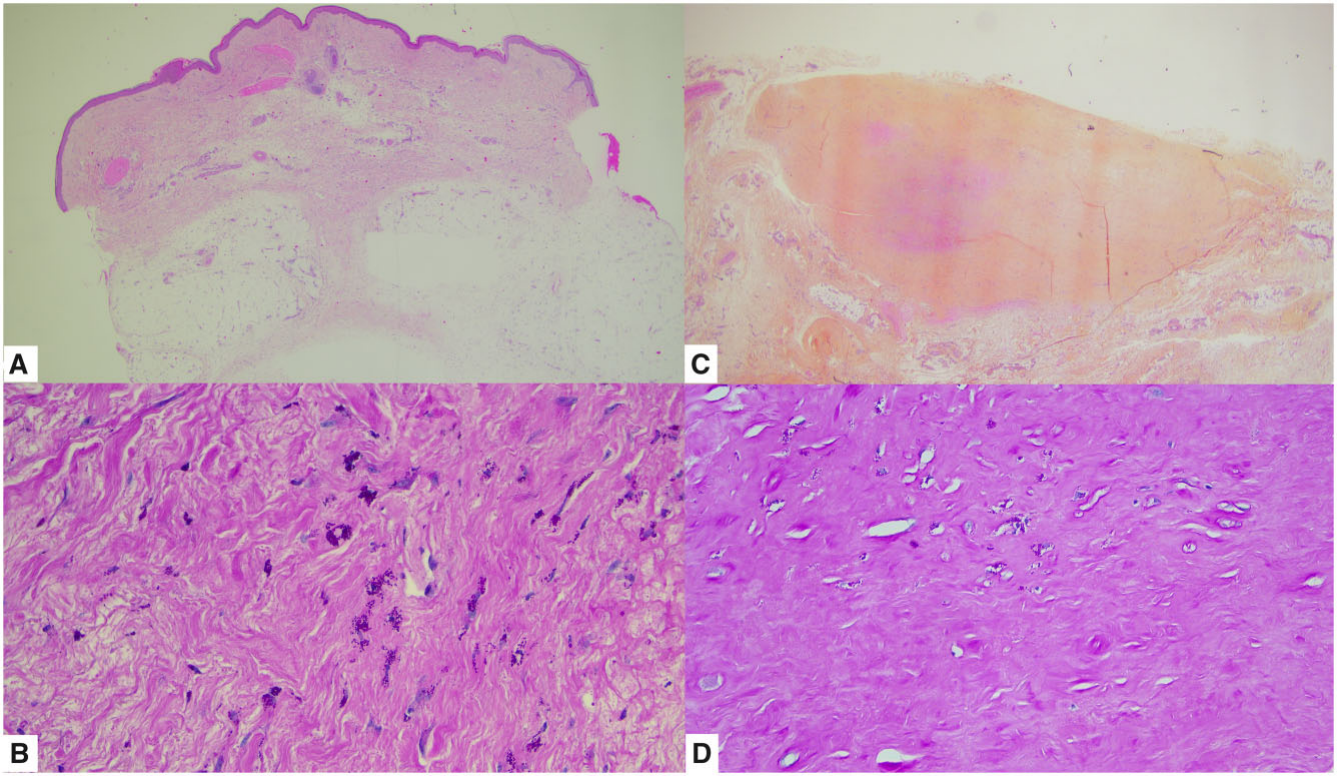
Pathological examination. **(A)** Deep skin biopsy showing a septal dermohypodermatitis [haematoxylin and eosin (H&E) stain, ×25). **(B)** Reactive fibrotic and necrobiotic subcutaneous nodule (H&E stain, ×50). **(C)** Skin biopsy and nodule showing many non-foamy macrophages (H&E stain, ×200). **(D)** Non-foamy macrophages whose cytoplasm contained multiple PAS-positive intracytoplasmic granulations, highly suggestive of *T. whipplei* (PAS stain, ×200)

The patient was treated with doxycycline 200 mg/day and plaquenil 600 mg/day, with good efficacy on the ankle arthritis, hypodermitis and oedema of the lower calves and breathlessness, and weight gain of 5 kg in 3 months. The removed nodule did not recur and the other nodules have decreased in size since the beginning of the treatment.

To our knowledge, this case is the first in the literature to describe a Whipple’s disease diagnosed in a patient with RF-positive polyarthritis and to describe a biopsy of pseudorheumatoid nodules revealing Whipple’s disease. Occurrence of RF in serum is rather unspecific and can be found in various morbid situations, including infections, as well as in healthy individuals. Various forms of nodules and articular involvements are common in patients with Whipple’s disease [1]. Polyarthritis in the patient could be considered as reactive to infection rather than a rheumatoid polyarthritis. It is known that treatment with immunomodulatory drugs, especially TNF inhibitors, can unmask Whipple’s disease. A recent case–control study revealed 19 publications of TNF inhibitor therapy used for seronegative polyarthritis in 41 patients in whom the diagnosis of Whipple’s disease was made later [2]. In total, 16 patients developed fever, 35 presented with arthritis and 15 had weight loss. Physiopathology suggests that immunomodulatory drugs significantly increase bacterial replication, apoptosis and macrophage repolarization [3]. Therefore it is suggested in many studies that Whipple’s disease should be suspected in patients showing resistance or frequent recurrence of chronic arthritis, if seronegative, who are under treatment with bDMARDs, especially in the presence of new, unexpected symptoms [4]. This case suggests that Whipple’s disease should be researched in patients with arthritis, even RF-positive and/or pseudorheumatoid nodules, especially if multiresistant to bDMARDs.

## Data Availability

The data underlying this article will be shared upon reasonable request to the corresponding author.
